# Supplementary material for: Identification of photoperiod-induced specific miRNAs in the adrenal glands of Sunite sheep (Ovis aries)
Source: Front Vet Sci. 2022 Jul 22;9:888207. doi: 10.3389/fvets.2022.888207 (PMC9354845; doi:10.3389/fvets.2022.888207)
Supplement: Supplementary file 1 [file Data_Sheet_1.ZIP › Supplementary file/miRDeep2 file.zip!/miRDeep2 file/Novel_Structure/LP42A1/Novel_670.pdf]

Provisional ID	:	NC_019481.2_33474
Score total	:	2794.8
Score for star read(s)	:	3.9
Score for read counts	:	2787.3
Score for mfe	:	2
Score for randfold	:	1.6
Score for cons. seed	:	
Total read count	:	5479
Mature read count	:	5437
Loop read count	:	0
Star read count	:	42

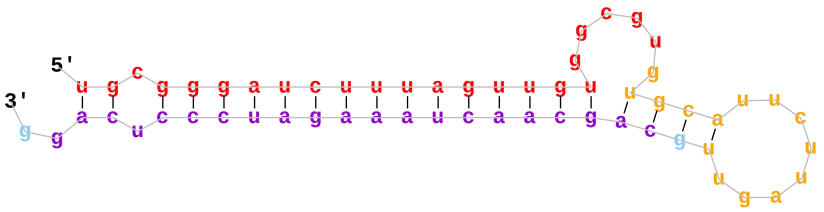

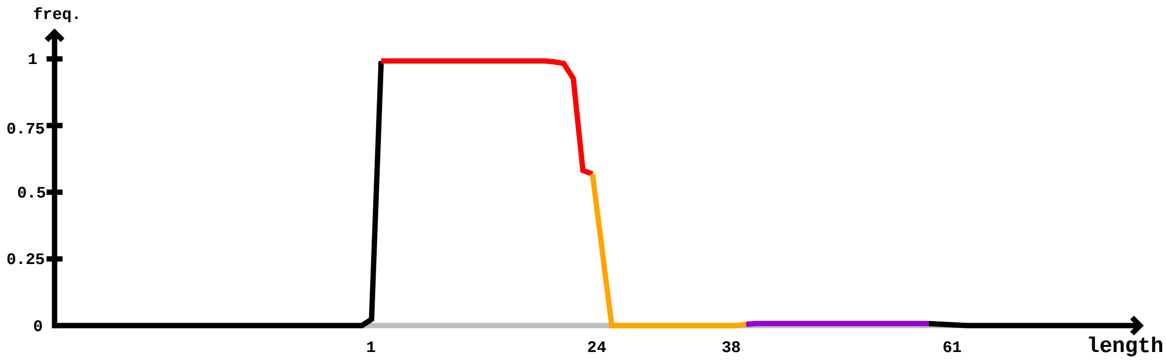


```
                                      Mature                              Star
5'- aauaacccauaaaagcagccgacguugcggcaugcgggaucuuuaguuguggcgugugcauucuuaguugcagcaacuaaagaucccucaggcugcaaugaagaucagaga  -3'  obs
    aauaacccauaaaagcagccgacguugcggcaugcgggaucuuuaguuguggcgugugcauucuuaguugcagcaacuaaagaucccucaggcugcaaugaagaucagaga       exp
    ...................(..(((((((((.((.(((((((((((((((......((((........))))))))))))))))))).)).)))))))))..)........  reads  mm  sample
    ...............................augcgggaucuuuaguugC.............................................................  1      1   seq
    ...............................augcgggaucuuuaguugu.............................................................  3      0   seq
    ...............................augcgggaucuuuaguuguA............................................................  1      1   seq
    ...............................augcgggaucuuuaguugug............................................................  8      0   seq
    ...............................augcgggaucuuuaguugugg...........................................................  23     0   seq
    ...............................augAgggaucuuuaguugugg...........................................................  2      1   seq
    ...............................augcCggaucuuuaguuguggc..........................................................  1      1   seq
    ...............................augcgggaucuuuaguuguggc..........................................................  48     0   seq
    ...............................augcgggaucuuuaguuguggU..........................................................  23     1   seq
    ...............................augAgggaucuuuaguuguggcg.........................................................  1      1   seq
    ...............................augcgggaucuuuaguuguggcA.........................................................  17     1   seq
    ...............................augcgggaucuuuaguuguggUgu........................................................  1      1   seq
    ...............................augcUggaucuuuaguuguggcgu........................................................  1      1   seq
    ................................ugcgggaucuuuaguugC.............................................................  12     1   seq
    ................................ugcgggaucuuuaguugA.............................................................  3      1   seq
    ................................ugcgggGucuuuaguugug............................................................  3      1   seq
    ................................ugcgggaucuuuaguuguA............................................................  18     1   seq
    ................................ugGgggaucuuuaguugug............................................................  1      1   seq
    ................................ugcAggaucuuuaguugug............................................................  2      1   seq
    ................................ugcCggaucuuuaguugug............................................................  1      1   seq
    ................................ugcgggaucuuuaguugugA...........................................................  193    1   seq
    ................................uAcgggaucuuuaguugugg...........................................................  5      1   seq
    ................................ugcCggaucuuuaguugugg...........................................................  12     1   seq
    ................................ugcgAgaucuuuaguugugg...........................................................  2      1   seq
    ................................ugcgggaucuuuaguugAgg...........................................................  1      1   seq
    ................................ugcgggaucuuuUguugugg...........................................................  1      1   seq
    ................................ugcgggaucuuuaguugugU...........................................................  52     1   seq
    ................................ugcgggaucuuuaguugCgg...........................................................  2      1   seq
    ................................ugcAggaucuuuaguugugg...........................................................  9      1   seq
    ................................ugcgggaucuuuaguuguAg...........................................................  2      1   seq
    ................................ugcgggaucuuuagCugugg...........................................................  2      1   seq
    ................................ugcgggaucuuuagCuguggc..........................................................  3      1   seq
    ................................ugcAggaucuuuaguuguggc..........................................................  8      1   seq
```

```
                                 Mature                               Star
aauaacccauaaaaagcagccgacguugcggcaugcgggaucuuuaguuguggcgugugcauucuuaguugcagcaacuaaagaucccucaggcugcaaugaagaucagaga
.................................ugUgggaucuuuaguuguggc..........................................................   4     1   seq
.................................ugcgAgaucuuuaguuguggc..........................................................   7     1   seq
.................................ugcgggaucuuuaguuguggU..........................................................   520   1   seq
.................................uAcgggaucuuuaguuguggc..........................................................   7     1   seq
.................................ugcgggaucuuuaguuguAgc..........................................................   1     1   seq
.................................ugcgggaucuuuaguugugUc..........................................................   1     1   seq
.................................ugcCggaucuuuaguuguggc..........................................................   22    1   seq
.................................ugcgggGucuuuaguuguggc..........................................................   3     1   seq
.................................ugcgggaucuuuaguuguggc..........................................................   1243  0   seq
.................................ugcgggaucuuuagCuguggcg.........................................................   5     1   seq
.................................ugcgggGucuuuaguuguggcg.........................................................   23    1   seq
.................................ugcUggaucuuuaguuguggcg.........................................................   1     1   seq
.................................uAcgggaucuuuaguuguggcg.........................................................   25    1   seq
.................................ugcgggaucuuuagCuguggcgu........................................................   5     1   seq
.................................ugcgggaucuuuaguuguggcgu........................................................   3016  0   seq
.................................ugcUggaucuuuaguuguggcgu........................................................   2     1   seq
.................................uAcgggaucuuuaguuguggcgu........................................................   12    1   seq
.................................ugcgggGucuuuaguuguggcgu........................................................   20    1   seq
.................................ugcAggaucuuuaguuguggcgu........................................................   21    1   seq
.................................ugcgAgaucuuuaguuguggcgu........................................................   12    1   seq
.................................ugcgggaucuuuaguuguggcgug.......................................................   23    0   seq
..................................gcgggaucuuuaguuguggcg.........................................................   2     0   seq
.......................................................................cCgcaacuaaagaucccuc......................   1     1   seq
.......................................................................cagcaacuaaagaucccucag....................   23    0   seq
.......................................................................cagcaacuaaagaucccucaggc..................   3     0   seq
........................................................................agcaacuaaagaucccucaggc..................   15    0   seq
```
